# Identification of a Potential Susceptibility Locus for Macular Telangiectasia Type 2

**DOI:** 10.1371/journal.pone.0024268

**Published:** 2012-08-31

**Authors:** Nancy L. Parmalee, Carl Schubert, Maria Figueroa, Alan C. Bird, Tunde Peto, Mark C. Gillies, Paul S. Bernstein, Krzysztof Kiryluk, Joseph D. Terwilliger, Rando Allikmets, for the MacTel Project

**Affiliations:** 1 Department of Ophthalmology, Columbia University, New York, New York, United States of America; 2 Department of Genetics and Development, Columbia University, New York, New York, United States of America; 3 Department of Medicine, Columbia University, New York, New York, United States of America; 4 Department of Psychiatry, Columbia University, New York, New York, United States of America; 5 Columbia Genome Center, Columbia University, New York, New York, United States of America; 6 Department of Pathology and Cell Biology, Columbia University, New York, New York, United States of America; 7 The EMMES Corporation, Rockville, Maryland, United States of America; 8 Moorfields Eye Hospital, London, United Kingdom; 9 Save Sight Institute, Department of Clinical Ophthalmology and Eye Health, The University of Sydney, Sydney, Australia; 10 Department of Ophthalmology and Visual Sciences, Moran Eye Center, University of Utah School of Medicine, Salt Lake City, Utah, United States of America; Innsbruck Medical University, Austria

## Abstract

Macular Telangiectasia type 2 (MacTel) is a relatively rare macular disease of adult onset presenting with distortions in the visual field and leading to progressive loss of visual acuity. For the purpose of a gene mapping study, several pedigrees were ascertained with multiple affected family members. Seventeen families with a total of 71 individuals (including 45 affected or possibly affected) were recruited at clinical centers in 7 countries under the auspices of the MacTel Project. The disease inheritance was consistent with autosomal dominant segregation with reduced penetrance. Genome-wide linkage analysis was performed, followed by analysis of recombination breakpoints. Linkage analysis identified a single peak with multi-point LOD score of 3.45 on chromosome 1 at 1q41-42 under a dominant model. Recombination mapping defined a minimal candidate region of 15.6 Mb, from 214.32 (rs1579634; 219.96 cM) to 229.92 Mb (rs7542797; 235.07 cM), encompassing the 1q41-42 linkage peak. Sanger sequencing of the top 14 positional candidates genes under the linkage peak revealed no causal variants in these pedigrees.

## Introduction

Macular telangiectasia is a group of diseases characterized by Gass and Blodi in 1993 [1] and reclassified by Yannuzzi in 2006 [2]. Macular telangiectasia type 2 (MacTel) generally presents bilaterally between the 5^th^ and 7^th^ decades of life with reduction in central vision and distortion in the visual field. The cause of the disease is unknown and there is no treatment.

Clinical characteristics of MacTel include loss of retinal transparency, autofluorescence changes in the macula, macular edema, presence of intraretinal crystals, and disruption of macular pigment transport. Symptoms of advanced disease include the presence of a macular hole, dilated and tortuous vessels in the perifoveal region, leakage from retinal vessels and neovascularization arising from the intraretinal vessels [3], [4], [5], [6], [7], 8,9,10,11,12. Patients experience distortions in central vision, including parafoveal scotoma, and metamorphopsia. Both genders are affected equally.

While MacTel had been presumed to be a very rare disease, recent epidemiological studies suggest that it is under-diagnosed and, therefore, more common than previously thought. The Beaver Dam Eye Study recently reported a prevalence of 0.1% in a retrospective study of 4,790 individuals, aged 43–86 years of age [13]. The Melbourne Collaborative Cohort estimated a probable prevalence of 0.0045% based on evaluation of 3,784 images where macular disease was noted, out of a study population of 22,415 participants [9]. Both studies used available population data where retinal images had been obtained to assess other macular diseases in populations. In both studies, however, images had not been taken with the intent to diagnose MacTel; therefore the authors concluded that subtle features of MacTel were likely missed without specialized imaging, such as fluorescein angiography and blue light reflectance imaging.

**Figure 1:**
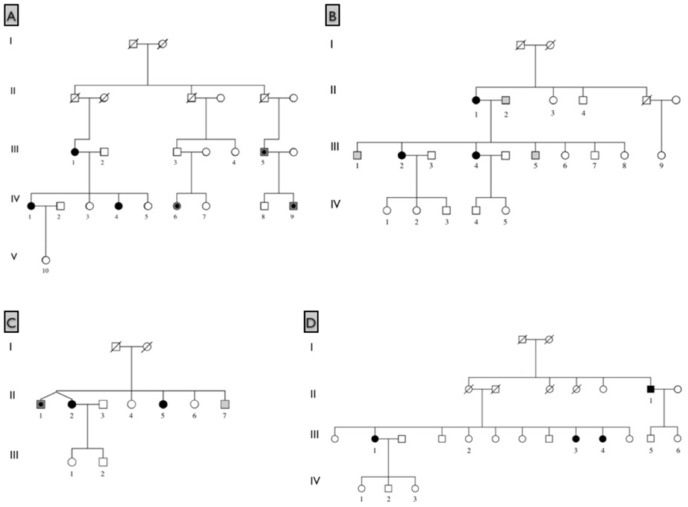
Four families with multiple relatives affected with MacTel. Black shaded symbols represent affected; dark gray shading represents possibly affected; light gray shading represents probably not affected; unshaded symbols represent unaffected or unexamined relatives. Numbered individuals were enrolled and examined.

MacTel was proposed to have a genetic component based on case reports of affected sibling pairs and concordant monozygotic twins [3], [14], [15], [16], [17], [18], [19]. To test the hypothesis that MacTel is an inherited disease, family members of probands were actively recruited and given full ophthalmic examinations. Gillies et al. [19] have previously reported four multiplex families included in this study. Additional multiplex families were subsequently identified, strengthening the hypothesis that variants in one or more genes underlie in the etiology of MacTel. Figure 1 shows four of the largest families identified with multiple relatives affected with MacTel.

The MacTel Project was established as a consortium of basic science researchers and clinicians in order to study the natural history, identify the cause(s) of the disease, and propose targets for treatment. Patients were screened and enrolled at 23 clinical centers in seven countries (Australia, Germany, France, the U.K., Israel, Switzerland, and the United States). Family members were actively recruited and given complete ophthalmic examinations. Seventeen multiplex families were identified that were informative for linkage analysis, together with additional parent-child duos Altogether, these data provided a basis for genome-wide linkage mapping that identified a significant linkage peak for this disease.

## Results

### Study population

Seventeen families with a total of 71 individuals (45 affected or possibly affected) were analyzed for linkage. The inheritance pattern in families with more than one affected individual was consistent with autosomal dominant transmission. MacTel exhibits reduced penetrance based on the observation that in some multiplex families neither parent is clearly affected with the disease. Variable disease expressivity is evident in many pedigrees in this cohort; while probands presented to the clinic experiencing vision loss, some relatives were given a diagnosis of MacTel only after a complete ophthalmic exam as a part of this study. Based on a masked analysis of images by a central reading center, not influenced by the initial diagnosis from a recruiting center, all subjects were categorized as definitely affected, possibly affected, probably not affected, or definitely not affected. This clearly illustrates the variable expressivity of MacTel, complicating genetic analysis. No gender bias was observed in patients, and male to female and female to male transmissions were both observed in the pedigrees. Most families are too small to make a reliable estimation of the ratio of affected offspring, however, four large families had ratios of affected offspring consistent with autosomal dominant inheritance.

### Multi-point linkage analysis

**Figure 2:**
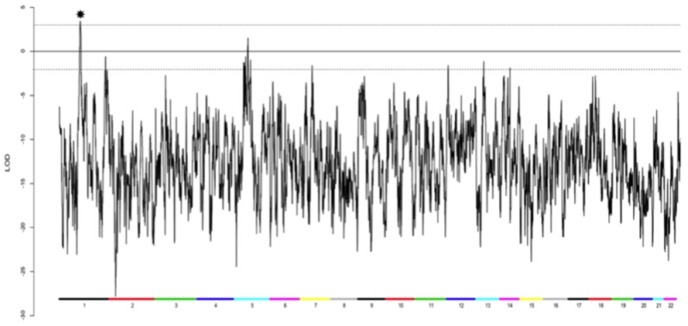
Genome-wide affected only linkage scan in 17 families with 71 individuals. Colored bars at the bottom of the figure label each chromosome. A maximum LOD of 3.45 score was observed at chromosome 1q41-42 (starred). A second region with a positive LOD = 1.52 was observed on chromosome 5. LOD scores for the remainder of the genome were negative.

**Figure 3:**
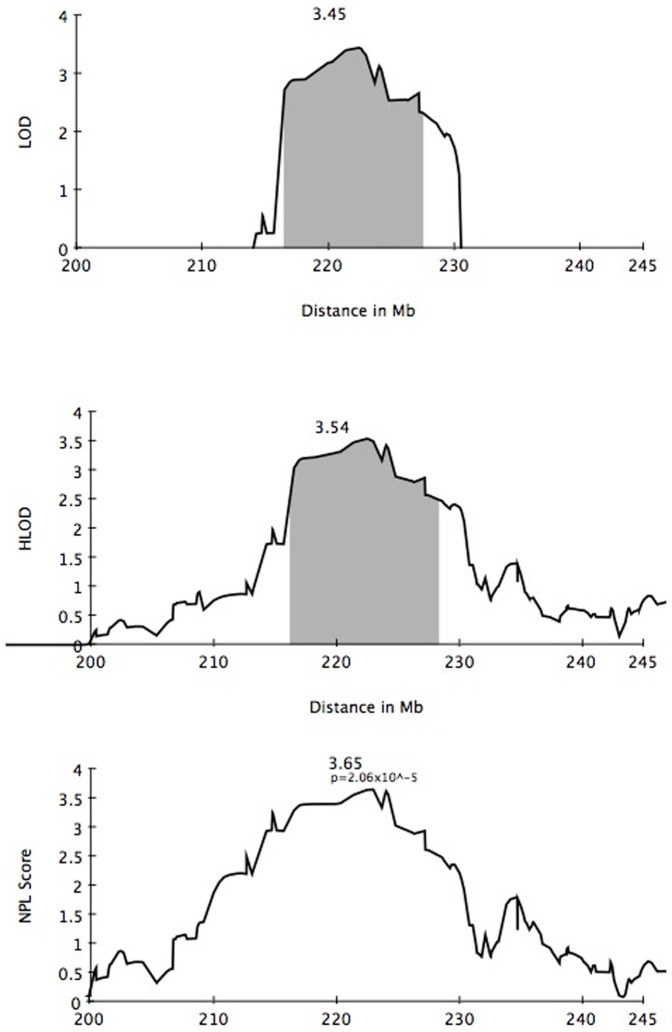
LOD, HLOD, and NPL scores for chromosome 1 in 17 families. The 1-LOD support interval around the maximum LOD of 3.45 is shaded in gray.

A total of 112 individuals in 33 MacTel families were screened on Illumina 1 M Duo arrays. Seventeen informative families were analyzed by multi-point, affected-only parametric linkage analysis under an autosomal dominant model using a subset of independent SNPs from the Illumina 1 M chip. Family members diagnosed as possibly and definitely affected were coded as affected. Relatives diagnosed as unaffected or probably not affected were coded as unknown. There was a single significant peak observed on chromosome 1, with a LOD score of 3.45 and HLOD of 3.54 (alpha = 0.93) over an interval of approximately 15 Mb (Figures 2 and 3). Only one other region, on chromosome 5, yielded a positive LOD score, spanning an interval of approximately 3.4 Mb (LOD = 1.52, HLOD = 2.43, alpha = 0.76). Ten families were linked to this region; two families were unlinked, and the remainder showed LOD scores close to zero. Ten regions, totaling 13.2 Mb, had negative LOD scores between −2 and 0; the remainder of the genome yielded LOD scores below −2, sufficiently negative for exclusion of linkage under the parameters assumed (Table 1). One large family (family 8) was split into two smaller families because of the large number of missing family members between the two branches. All families were linked to the peak on chromosome 1, with the exception of one branch of this large family. The individuals coded as affected in the unlinked branch all had a diagnosis of “possibly affected.”

**Table 1:** Exclusion based on parametric LOD score less than −2 under an autosomal dominant model.

Chromosome	Regions not excluded by parametric linkage (Mb)	Regions not excluded by allele sharing (Mb)
1	1q41 213.2 (2 markers)	1q32.2 205.89–208.95
	1q41-42 214.27–230.038	1q41-42 214.32–229.92
	(LOD = 3.43, 15.768 Mb)	
2	-	2q12-23.3 134.49–149.84
		2q43-44 240.24–243.12
3	-	-
4	-	4q22.1-24 86.68–99.52
5	5q14.3 87.1–88.46 (1.36)	
	5q15-21.196.85-97.71 (0.86)	
	5q21.3-23.1 109.26–111.17 (1.91)	
	5q23.1 116.81–119.55 (2.74)	
	5q32-33.1 146.41–149.78 (LOD = 1.52 alpha = 0.76; HLOD = 2.43, 3.37 Mb)	
	5q33.1-33.2 150.46–152.95 (2.49)	
	5q35.2 172.63–172.69 (0.06)	
6	-	-
7	7q33 136.74–137.33(0.59)	7p14.3-q21.3 33.75–77.87
		7q31.33-35 125.92–145.41
8	-	-
9	-	8p23.1 8.10–11.76
		8q21.12-21.2 78.0–86.35
10	-	-
11	-	11p15.4-15.1 7.0–16.89
12	12p13.31 6.62–7.16 (0.54)	12p13.31 5.38–7.03
13	13q31.1 80.56–83.03 (2.47)	-
14	14q32.33 106.14–106.36 (0.22)	14q31.2-32.13 82.66–94.26
		14q32.32-32.33 102.52–106.38
15	-	-
16	-	16p13.13 10.31–11.74
17	-	-
18	-	-
19	-	-
20	-	-
21	-	-
22	-	-

### Recombination mapping by determination of IBD allele sharing

**Figure 4:**
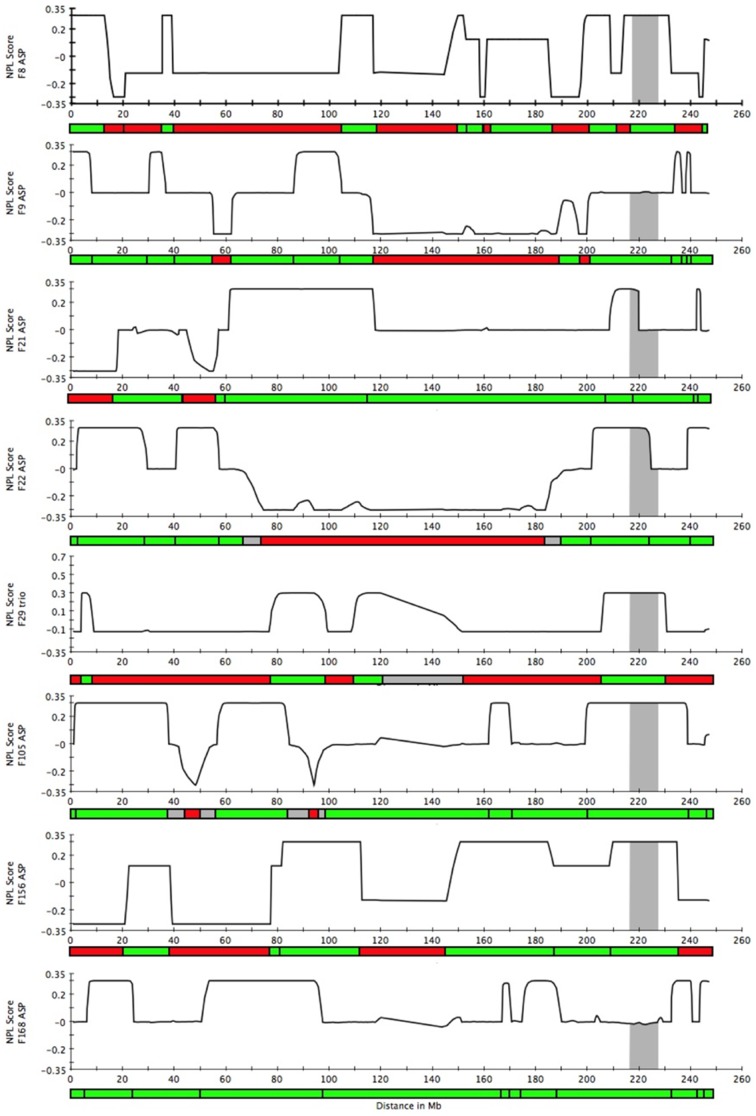
NPL scores indicating IBD allele sharing on chromosome 1 in 8 families. Recombinations were mapped by determining IBD allele sharing in 8 families. Regions where at least one allele is shared IBD are marked in green; regions excluded by virtue of no alleles shared IBD are marked in red. Gray bars represent the 1-LOD support interval. For ASPs with parental genotypes missing, 0.3 = 2 alleles shared, 0 = 1 allele shared, −0.3 = 0 alleles shared. For ASPs with parents genotyped and one parent affected, 0.3 = 2 alleles shared, 0.12 = 1 allele shared from the affected parent, −0.12 = 1 allele shared from the unaffected parent, −0.3 = 0 alleles shared. For the affected sib trio, 0.6 = 2 alleles shared between all sibs, 0.3 = 1 allele shared between all sibs, and −.12 = 0 alleles shared between all sibs.

**Figure 5:**
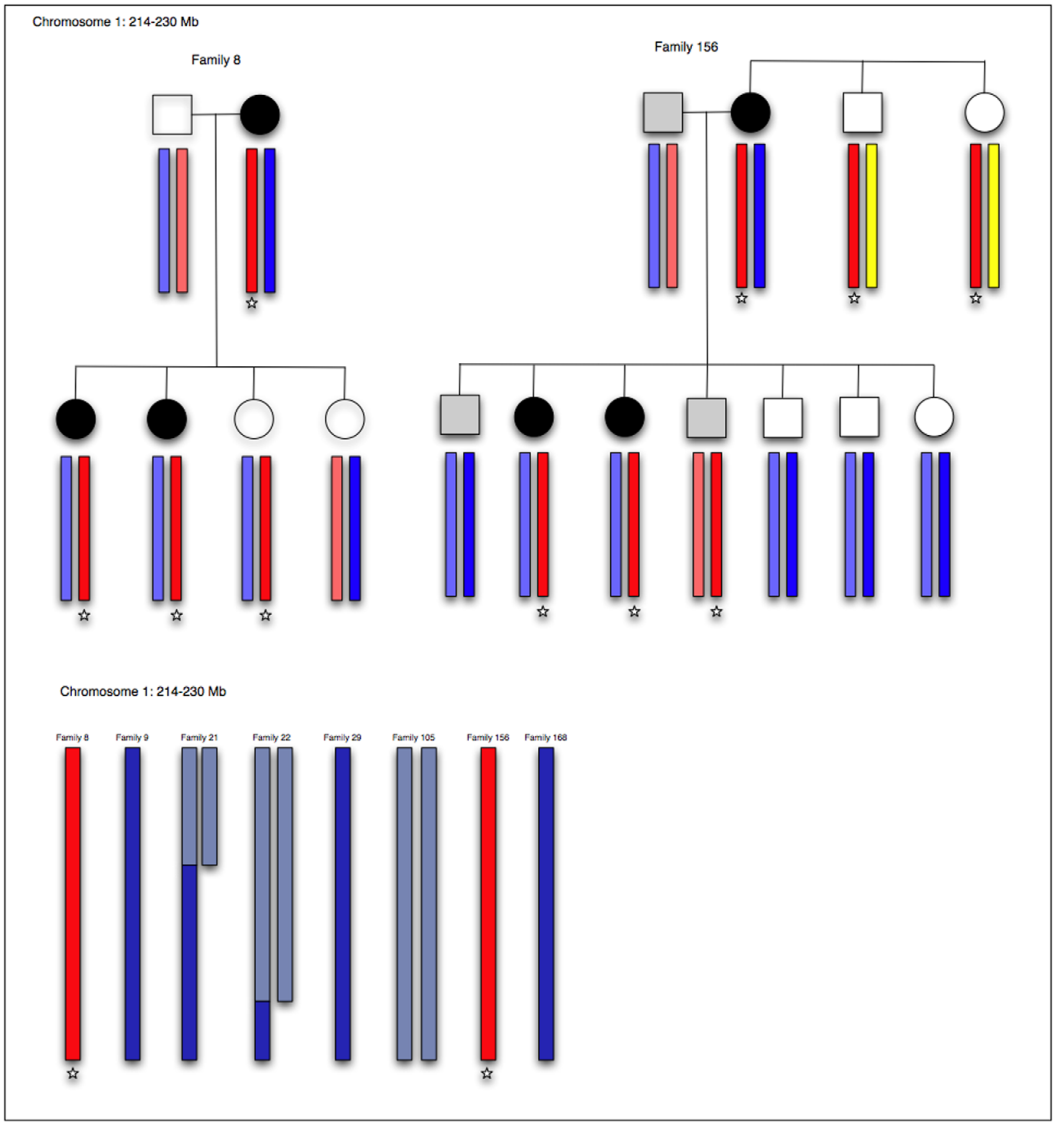
Segregating haplotypes and IBD allele sharing. Putative risk haplotypes (shown in red and designated with a star) are shown for two families where the parents in both families were genotyped and the phased haplotypes were resolved. Allele sharing identical by descent is shown for six additional families (five ASPs and one affected sibling trio). Phased haplotypes were not resolved in families where parental genotypes were missing. Regions with one allele shared IBD are shown in dark blue; regions with two alleles shared IBD are shown in purple.

IBD status was inferred along each chromosome with MERLIN [20], and only those positions where all affected individuals within a pedigree shared the same chromosomal segment were deemed consistent with the hypothesis of a necessary rare variant being located in a given genomic region. The full genome was analyzed for one trio of siblings and seven affected sib pairs, of which two had both parents genotyped (one parent in each family was affected). The results from the 8 families included in this analysis were then combined and the full genome was analyzed to map regions where exclusion was declared for at least one family; these regions were marked as excluded (Figure 4). Twelve chromosomes (3, 5, 6, 8, 10, 13, 15, 18, 19, 20, 21, and 22) were entirely excluded (Table 1). Chromosomes 1, 2, 4, 7, 9, 11, 12, 14, and 16 were partially excluded. In total, 153.81 Mb remained as potentially able to harbor a causal allele for MacTel under the assumption that a single copy of a single variant was necessary (but not sufficient) for disease (Table 1). Phased haplotypes were resolved in families 8 and 156, where both parents in each family were genotyped (Figure 5). The shared haplotypes in these families were consistent with IBD allele sharing.

### Comparison of IBD to linkage exclusion

Comparison of regions that were not excluded by either linkage analysis or by recombination breakpoint analysis revealed four regions that were not excluded by either analysis, including the significant linkage peak on chromosome 1. Three additional regions with LOD scores between 0 and −2 under autosomal dominant linkage analysis were not excluded by breakpoint analysis in strictly affected siblings: chromosome 7, 125.92–145.41 Mb; chromosome 12, 5.38–7.03 Mb; and chromosome 14, 102.52–106.38 Mb (positions based on recombination analysis). Reviewing regions of exclusion in these two different ways serves to clarify whether a chromosomal segment is excluded based on information from definitely affected individuals or possibly affected family members where the phenotype is less strictly defined. A total of 32.378 Mb were not excluded by parametric linkage analysis, including the two regions of positive linkage. A total of 153.81 Mb were not excluded by the more stringent recombination breakpoint analysis based only on definitely affected siblings.

### Sequencing of Positional Candidate Genes

A 1.8 Mb region of the 15.6 Mb chromosome 1 linkage interval from 221,168,406–222,994,872, corresponding to the maximum LOD score, was selected for sequencing, which included thirteen genes and one micro-RNA. This region was selected as a starting point in screening the interval. Efforts to sequence the remaining genes in the region are ongoing. All exons and flanking intronic regions of *DISP1*, *TLR5*, *SUSD4*, *BEND5*, *CAPN8*, *CAPN2*, *TP53BP2*, *FBXO28*, *DEGS1*, *NVL*, *CNIH4*, *WDR26*, and *CNIH3* genes and the micro-RNA, *MIR320B2* were screened by Sanger sequencing in two affected family members, one from family 8 (individual IV4), the other from family 156 (individual III2). Each of these families consists of an affected sib pair, unaffected siblings, and two parents, one of which is affected in each family. Altogether, sixteen variants (confirmed by bi-directional sequencing) were detected in coding regions of these genes (Table 2). Six of these were synonymous variants; of the 10 non-synonymous variants, 5 were frequent polymorphisms, 2 were known variants of low or undetermined minor allele frequency that failed to segregate with the disease, and 2 were previously unknown missense variants that failed to segregate with the disease. One known variant with MAF = 0.033, p.Val404Ile in NVL, was detected in one family, in which it was present in all affected family members, one possibly affected sibling, and no unaffected family members. However, this variant is more frequent than would be expected given the prevalence of MacTel and, therefore, not deemed disease-associated.

**Table 2:** Variants detected by Sanger sequencing in two affected individuals.

Gene	Number of exons	Variants detected	MAF	Notes
*DISP1*	7	Unknown c.75G>A p.Pro25Pro		Syn
		Unknown c.2835A>G p.Lys945Lys		Syn
		rs9441941 c.3822A>C p.Pro1274Pro	.292	Syn
*TLR5*	1	rs5744174 c.1846T>C p.Phe616Leu	.375	FV
		rs2072494 c.1775A>G p.Asn592Ser	.139	FV
*SUSD4*	9	None		
*BEND5*	1	Unknown c.986G>A p.Gly329Asp		DNS
*CAPN8*	19	rs35539373 c.734C>A p.Ser245Tyr	.472	FV
		rs61823553 c.1775C>T p.Thr592Met	.389	FV
*CAPN2*	21	rs17599 c.1702A>C p.Lys568Gln	.292	FV
		Unknown c.582G>A p.Ala194Ala		Syn
*TP53BP2* (1, 12)	18	rs61749337 c.566C>T p.Ala189Val	ND	DNS
		rs34683843 c.685C>A p.Gln229Lys	.058	DNS
*FBXO28*	5	None		
*DEGS1* (1)	3	None		
*NVL* (15, 22, 23)	23	rs7534447 c.456G>A p.Arg152Arg	.058	Syn
		rs3754090 c.738G>A p.Leu246Leu	.058	Syn
		rs34631151 c.1210G>A p.Val404Ile	.033	
*CNIH4* (3)	5	Unknown c.48T>G het p.Phe6Val		DNS
*WDR26*	13	None		
*MIR320B2*	1	None		
*CNIH3*	6	None		

Syn = synonymous.

FV = frequent variant.

DNS = does not segregate with disease.

( ) indicates exons that could not be sequenced.

## Discussion

This study presents the first genome-wide linkage analysis of MacTel. The identification of multiplex families with the disease supports the hypothesis of a genetic component to the disease. Previously, we investigated candidate genes by Sanger sequencing in the same cohort; however, no variants associated with MacTel were detected [21].

We examined 17 multiplex families in a genome-wide linkage scan and detected significant linkage to 1q41-42, with a multipoint LOD score of 3.45. Assuming an autosomal dominant pattern of inheritance, and analyzing only affected and possibly affected individuals, all families were linked to this locus, with the exception of one branch of a large family, analyzed separately. Two relatives in the unlinked branch of family 1 were diagnosed as “possibly affected.” One possibility is that this family is segregating a risk allele at a different locus; another possibility is that the two relatives are not actually affected with the disease. The detection of a significant linkage peak provides the first evidence of a susceptibility locus for this disease.

Analysis of recombination breakpoints in strictly affected siblings defined chromosomal segments that were incompatible with inheritance of a rare disease allele. The results from individual families were combined to assess which regions across the genome are not excluded in any family, as would be expected if MacTel is a monogenic disease, caused by variants in the same region in all families. Two regions on chromosome 1 were compatible with monogenic, autosomal dominant inheritance: one region corresponds to the region of the linkage peak; the other, smaller region is 5.3 Mb centromeric to the boundary of the linkage region. A second region of positive linkage on chromosome 5, with alpha = 0.76, yielded LOD scores below the threshold for significance. This region was excluded in one family based on the observation of 0 alleles shared between affected siblings. This analysis was limited to affected sibling pairs and one sibling trio that were diagnosed as definitively affected. Family members diagnosed as “possibly affected” were excluded from these analyses. This, more stringent, use of affected status was applied because the output of this analysis is a binary classification of either included or excluded, therefore the penalty for including a family member with an incorrect diagnosis is higher than for parametric linkage analysis.

MacTel was previously believed to be a disease with no discernable pattern of inheritance; however, in many families identified in this study, the disease appears to segregate as a monogenic, autosomal dominant trait. This does not preclude the possibility of genetic heterogeneity, but rather provides a starting point for genetic dissection of the trait in families. Intensive efforts have been undertaken by collaborators within the MacTel project to refine the definition of the phenotype and gain insight into the progression of MacTel. It is unknown at this point whether family members diagnosed as “possibly affected” are in the early stages of the disease and will eventually manifest full signs of MacTel, or whether these individuals carry modifiers that lessen the expression of the phenotype. It is noted that the median age of relatives diagnosed as possibly affected is younger than those relatives with a definite diagnosis. For the linkage analysis part of this study, we categorized “possibly affected” relatives as affected, given that they exhibit signs of the diseases that are not typically seen in the unaffected population. While the ratios of affected to unaffected individuals in large families correspond well to the expected ratios for a Mendelian trait with autosomal dominant inheritance, most families in the cohort are not large and, in most cases, parents are deceased or unavailable for screening due to the late age of onset of the disease. The observation that in some pedigrees parents of affected offspring are unaffected has suggested that MacTel is not fully penetrant. Whether this is due to locus heterogeneity, the presence of modifying alleles segregating in families, or environmental influences acting on an underlying genetic predisposition is unknown at this time. The chromosome 1 linkage region with a significant LOD score is the primary region of interest for a causative variant for MacTel. The second region of positive linkage, on chromosome 5, was excluded in the combined IBD analysis of affected siblings. Future work will include complete sequencing of the linkage region on chromosome 1, as well as other regions not excluded by IBD analyses, and analysis of additional families as they become available.

## Materials and Methods

### Study cohort

Patients, relatives, and controls were recruited at 23 participating clinical centers in 7 countries (Australia, Germany, France, The United Kingdom, Switzerland, Israel, and the United States). Informed written consent was obtained at each participating clinical center in accordance with ethics protocols for human subjects approved by the appropriate governing body at each site in accordance with the Declaration of Helsinki. All protocols and records of consent were centrally managed by the EMMES Corporation (Rockville, Maryland). The following ethics boards granted approval for human subjects enrollment at each participating center. Quinze-Vingts, Paris, France: Comite De Protection Des Personnes Hopital Saint-Antonie; Centre for Eye Research, Victoria, Australia: The Royal Victorian Eye & Ear Hospital; Clinique Ophtalmolgie de Creteil, Paris, France: Comite De Protection Des Personnes Hopital Saint-Antonie; Hospital Lariboisiere, Paris, France: Comite De Protection Des Personnes Hopital Saint-Antonie ; Jules Stein Eye Institute, UCLA, Los Angeles, United States: The UCLA Institutional Review Board; Lions Eye Institute, Nedlands, Australia: Sire Charles Gairdner Group Human Research Ethics Committee; Manhattan Eye, Ear & Throat Hospital, New York, United States: Lenox Hill Hospital Institutional Review Board; Moorfields Eye Hospital, London, U.K.: National Research Ethics Service; Retina Associates of Cleveland, Inc., Cleveland, United States: Sterling Institutional Review Board; Save Sight Institute, Sydney, Australia: South Eastern Sydney Illawarra Area Health Service Human Research Ethics Committee – Northern Hospital Network; Scripps Research Institute, La Jolla, United States: Scripps Institutional Review Board; St. Franziskus Hospital, Munster, Germany: Ethik-Kommission Der Arztekammer Westfalen-Lippe Und der Medizinishchen Fakultat der Westfallschen Wilhelms-Universitat; The Goldschleger Eye Institute, Tel Hashomer, Israel: Ethics Committee The Chaim Sheba Medical Center; The New York Eye and Ear Infirmary, New York, United States: The Institutional Review Board of the New York Eye and Ear Infirmary; The Retina Group of Washington, Olympia, United States: Western Institutional Review Board; University of Bonn, Bonn, Germany: Rheinische Friedrich-Wilhelms-Universitat Ethik-Kommission; University of Chicago, Chicago, United States: The University of Chicago Division of Biological Sciences – The Pritzker School Institutional Review Board; University of Michigan, Ann Arbor, United States: Medical School Institutional Review Board (IRBMED); University of Wisconsin, Madison, United States: Office of Clinical Trials University of Wisconsin School of Medicine and Public Health; The Wilmer Eye Institute of Johns Hopkins University, Baltimore, Maryland: Johns Hopkins School of Medicine Office of Human Subjects Research; Scheie Eye Institute University of Pennsylvania, Philadelphia, United States: University of Pennsylvania Office of Regulatory Affairs; University of Bern, Bern, Switzerland: Kantonale Ethikkommission Bern; John Moran Eye University of Utah, Salt Lake City, United States: The University of Utah Institutional Review Board; Bascom Palmer Eye Institute University of Miami, Miami, United States: The University of Miami Human Subjects Research Office; Columbia University, New York, United States: Columbia University Medical Center Institutional Review Board Category 4 waiver for research involving specimens obtained from de-identified subjects.

Participants were given a standardized ophthalmic examination, including best corrected visual acuity, fundus photography, fluorescein angiography, optical coherence tomography, and blue light reflectance. Images were adjudicated at the Reading Center at Moorfields Eye Hospital, London. Diagnoses were made in accordance with the criteria described by Clemons et al. [22] based on Gass and Blodi [1]. Retinal images were assessed for loss of transparency in the perifoveal region, dilated and telangiectatic blood vessels, especially in the temporal retina, and crystalline deposits. Each sample was assigned to one of four diagnostic categories: affected, possibly affected, probably not affected, or unaffected. Participants are re-evaluated at regular intervals over the course of the study.

Peripheral venous blood was drawn from each participant and used to isolate DNA (Qiagen blood maxi kit 51194, Valencia, CA). DNA concentration was determined using the NanoDrop spectrophotometer (Thermo Scientific, Wilmington, DE). DNA samples of low purity were subjected to column purification (Qiagen blood and tissue kit 69504).

### Genotyping and marker selection

Samples were genotyped on the Illumina 1 M chip. The linkage marker set was selected by pruning stringently for genotype quality in GenomeStudio using the following parameters: GenTrain threshold ≥0.50, cluster separation ≥0.16, number of no calls = 0. The remaining markers were pruned based on LD in PLINK [23], [24] to remove markers with r^2^ greater than 0.17. The final marker set consisted of 11,676 independent markers. An identity by state-based relatedness analysis was performed in PLINK using genome-wide marker sets to confirm family structures.

### Linkage Analysis

Parametric multipoint linkage analysis was carried out using MERLIN [20], with risk allele frequency of 0.001 and phenocopy rate of 0 under the assumption that for a rare disease, affected individuals in a multiplex family are most likely affected for the same genetic reason. Affected only analysis was performed, with unaffected individuals coded as unknown. Possibly affected family members were coded as affected, under the rationale that they exhibited specific phenotypic signs consistent with MacTel, though not sufficient for a definitive clinical diagnosis. Allele frequencies were estimated from a total of 112 individuals. Heterozygosity LOD scores and percentage of families linked to a locus (alpha) were calculated by MERLIN. As a confirmatory analysis that is less sensitive to model misspecification, we also performed a non-parametric linkage (NPL) analysis using MERLIN. Phased haplotypes were resolved in MERLIN for two families in which both parents were genotyped.

Recombinations were mapped by determining IBD allele sharing using the NPL algorithm in MERLIN. Only definitively affected sib pairs and trios were included in the initial analysis to mitigate the possibility of excluding chromosomal regions based on incorrect diagnoses. Allele sharing was determined by comparing NPL scores from siblings to scores from a simulated dataset, to determine the values associated with each allele sharing state. Where parental genotypes were missing, segments with NPL scores of −0.3 (0 alleles shared) were excluded. Where parents were genotyped and one parent was affected, segments with NPL scores of −0.3 (0 alleles shared) and −0.12 (1 allele shared from the unaffected parent) were excluded. For sib trios, segments with NPL scores of −0.12 were excluded (0 alleles shared among all three sibs). Chromosomal segments with intermediate values were classified as ambiguous. The results of this analysis were aggregated to compile a genome-wide map of chromosomal segments where at least one allele was shared IBD in siblings in all families. This result was compared to parametric linkage results from the entire cohort to examine allele sharing in the linkage interval on chromosome 1, to validate exclusion by negative parametric LOD score, and to search for regions that could be prioritized for gene screening by Sanger sequencing.

### Candidate gene sequencing

Genomic DNA from two unrelated affected individuals was amplified using primers specific for exons in the genes of interest. PCR amplification and sequencing was performed as previously described [21]. Sequencing was performed by Genewiz (South Plainfield, NJ). Primers were selected using Primer3 (http://frodo.wi.mit.edu/primer3/). Sequences were compared to the hg19 reference sequence.
